# Evolution of disaster preparedness studies: A bibliometric approach to exploring research trends and directions

**DOI:** 10.4102/jamba.v17i1.1800

**Published:** 2025-03-26

**Authors:** Rohana Rohana, Yusni Arni, Lukman Hakim, Elsi A. Fitri

**Affiliations:** 1 Mathematic Education Study Program, Faculty of Teacher Training and Education, Universitas PGRI Palembang, Palembang, Indonesia; 2 Elementary School Teacher Education Study Program, Faculty of Teacher Training and Education, Universitas PGRI Palembang, Palembang, Indonesia; 3 Natural Science Education Study Program, Faculty of Teacher Training and Education, Universitas PGRI Palembang, Palembang, Indonesia

**Keywords:** disaster preparedness, bibliometric analysis, research trends, evolution, research directions

## Abstract

This study examined trends in disaster preparedness literature, identified key current issues, and discovered future research avenues. This research adopts bibliometric analysis, VOSviewer 16.20 version and Biblioshiny. The results indicated some notable findings related to the development of disaster preparedness studies. This study successfully identified the most prominent trends in the literature of disaster preparedness, including the most commonly discussed and collaborative work among researchers in this discipline through the bibliometric approach. This research also highlighted that there has been a shifting focus in disaster preparedness studies over time, with an emphasis on aspects such as disaster risk mitigation and disaster risk reduction advocacy. In addition, this study seeks to identify the gaps in the research that has already been undertaken and provide possible issues for future research to fill these gaps and enhance the understanding of disaster preparedness. Because of the comprehensive nature of bibliometric analysis, this study could help scholars gain a broad view of relevant issues, while identifying prominent publications that are worthy of reference. The analysis provides insights into developments and innovations in disaster preparedness studies and identifies research gaps that require further exploration.

**Contribution:**

This study reveals trends and gaps and provides possible issues for future research in disaster preparedness, providing essential guideposts for future research and policy.

## Introduction

Disaster preparedness is an essential aspect of disaster risk management aimed at minimising the negative consequences of disasters (Amin Hosaini & Izadkhah 2020; Räsänen et al. 2020;). These disasters can include earthquakes, floods and tsunamis, which frequently cause significant damages to both human lives and infrastructure (Alem et al. 2021; Ma et al. 2021). Therefore, disaster preparedness has become a prominent focus among governments, international organisations and communities to decrease risks and enhance resilience (Ali Shah et al. 2020; Ayeb-Karlsson et al. 2019; Kılıç & Şimşek 2019; Manyena, Machingura & O’Keefe 2019; Shan et al. 2019).

The study of disaster preparedness has significantly expanded in recent years (Husna et al. 2022; Saja et al. 2019). This is similar to the study regarding the importance of disaster risk financing and societal preparedness in disaster-prone regions, especially in Indonesia, in which attempts have been made to enhance disaster management through several approaches (Marlina, Ruslanjari & Hakim 2024; Pamungkas et al. 2023). A number of studies have demonstrated that the involvement of society in disaster preparedness significantly boosted the effectiveness of disaster mitigation strategies (Wulandari et al. 2023). The correlation between knowledge and societal preparedness in disaster response is also an important factor affecting the efficiency of mitigation strategies (Firdaus et al. 2023). Considering previous studies, there are significant disparities in our understanding of numerous aspects of disaster preparedness, notably technological innovation, mitigation strategies and socio-economic dynamics (Bozkurt et al. 2020; Chang et al. 2021; Khan 2022; Patel et al. 2023). Therefore, this study aims to analyse the development of studies on disaster preparedness by applying a bibliometric approach with Biblioshiny and VOSviewer applications. This study analysed several articles regarding disaster preparedness from 2019 to 2023. Previously, several studies concerning individual disaster preparedness had already been done by Baker, Alamri and Aboshaiqah (2019) and Lye and Koh (2014). It is possible to identify research trends, the most proactive authors and ongoing collaborations between researchers and institutions through bibliometric analysis (Hoque, Haque & Islam 2022; Molassiotis et al. 2021; Rana 2022).

Bibliometrics is a quantitative analysis method employed to investigate the research development in a particular domain by determining publication trends, inter-author collaboration and mapping of knowledge network (Gaviria-Marin, Merigó & Baier-Fuentes 2019; Linnenluecke, Marrone & Singh 2020). In the context of disaster preparedness study, the use of bibliometrics enables us to comprehend the pattern and development trend of research, recognise influential authors and institutions, and examine ongoing topics in the discipline (Chang et al. 2021; Donthu et al. 2021). It is not only a holistic representation of the scientific establishment but also an extremely important guideline for future studies.

This study aimed to examine the phenomenon of disaster preparedness studies by adopting bibliometric methods. The data were gathered from prominent databases including Scopus (Elliott 2020; Higuchi 2021), and the researchers were evaluated based on the number of publications, their collaboration patterns, and the main topics addressed in the literature (Beatty, Shimshack & Volpe 2019; Clement et al. 2018; Mullis & Martin 2019). In addition, this study also provides meaningful insights regarding the ways in which disaster preparedness has been addressed in a wide range of geographical and temporal contexts (Lee et al. 2020; Zhang et al. 2021).

The results of this bibliometric analysis are expected to significantly contribute to understanding the development of disaster preparedness studies (Ariyachandra & Wedawatta 2023; Durrant et al. 2022). The findings acquired can be employed as a basis for decision-making by policymakers, academics and practitioners to enhance disaster preparedness strategies (Kramer 2017; Le Duff et al. 2020; Zuccaro, Leone & Martucci 2020). Hence, this study not only contributes to the scientific literature but also has practical implications for more effective disaster mitigation attempts.

## Research methods and design

Bibliometric studies are concerned with combining different structures, methods and instruments to study and investigate large amounts of scientific data while producing high-impact research (Donthu et al. 2021; Lozano et al. 2017).

Bibliometrics enables researchers to examine research trends in the data based on specific keywords and visualise both emerging trends and knowledge distribution in detail (Fletcher et al. 2018; Guo et al. 2022). Furthermore, bibliometric studies highlight an exhaustive overview, identify gaps in specific research, elicit research novelty and localise research in specific research areas (Donthu et al. 2021; Elamin et al. 2018; Nappo et al. 2021). Moreover, a bibliometric study of the literature on policy enables researchers to reveal fundamental policy shifts and policy tracks (Hu et al. 2018).

This research applied bibliometric methods and content analysis techniques. Bibliometric methods can be defined as studies that assist in examining the prevailing trends and inform future studies (Goyal & Kumar 2021; Seruni et al. 2019). Bibliometric methods incorporate text analysis, citation analysis, content analysis, keyword occurrence, co-cite analysis and co-authoring analysis (Barnes et al. 2019; Macmuda et al. 2022). Bibliometric analysis is closely related to the word metrics in general and scientific metrics more specifically (Purbanto & Hidayat 2023).

A general analogy is webometrics, which explores many aspects of the web (Ellegaard & Wallin 2015). This research investigates disaster preparedness by examining the data from the Scopus from 2019 to 2023. The data were assessed through Shiny’s bibliometric mapping software, VOSviewer. The investigation was carried out on 19 June 2024 by employing terminologies associated with disaster preparedness.

**FIGURE 1:**
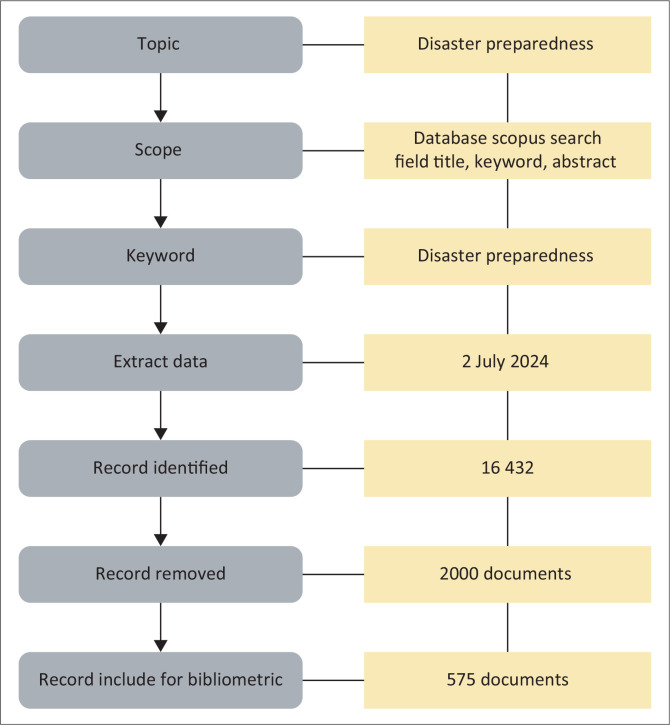
Flowchart of research method.

These keywords serve as the basis of journal lookups in electronic databases. The data from Scopus are a trusted source of peer-reviewed scientific publications with verified high-quality information (Almutairi, Mourshed & Ameen 2020; Rad, Mojtahedi & Ostwald 2021; Sakurai & Murayama 2019; Zhang et al. 2019). The bibliometric methods reflect the literature evolution on preparedness. Figure 1 illustrates the research workflow in a diagram.

The preliminary search for the phrase ‘Disaster preparedness’ yielded 16 432 publications categorised by subject area. These results were refined based on the research inclusion criteria and eliminated the duplicate papers. The revised results were saved as CSV files for subsequent processing. Selected articles yielded 572 articles that were examined using a bibliometric approach. The following are the research questions:

- **RQ1:** What are the disaster preparedness research trends regarding the number of publications yearly?
- **RQ2:** What are the top 10 journals that have been publishing disaster preparedness research?
- **RQ3:** Who are the top 10 authors contributing the highest amount to disaster preparedness research?
- **RQ4:** What are the top 10 countries contributing the most to disaster preparedness research?
- **RQ5:** Which countries are most actively publishing research on disaster preparedness, and how are their collaboration networks?
- **RQ6:** What are the most consequential topics in disaster preparedness?
- **RQ7:** What are the trending disaster preparedness topics?
- **RQ8:** What are the most potential topics for disaster preparedness research in future?
- **RQ9:** What institutions or organisations frequently appear in disaster preparedness articles?
- **RQ10:** What is the best methodology to explain the evolution of disaster preparedness over time?

### Ethical considerations

This article followed all ethical standards for research without direct contact with human or animal subjects.

## Results

This study examined publication trends in disaster preparedness literature and identified authors who are contributing to this research area. A bibliometric analysis was conducted to maximally visualise the frequently appearing words, highlighting the topics researched from previous years to the present. The bibliometric analysis was mapped differently.

**FIGURE 2:**
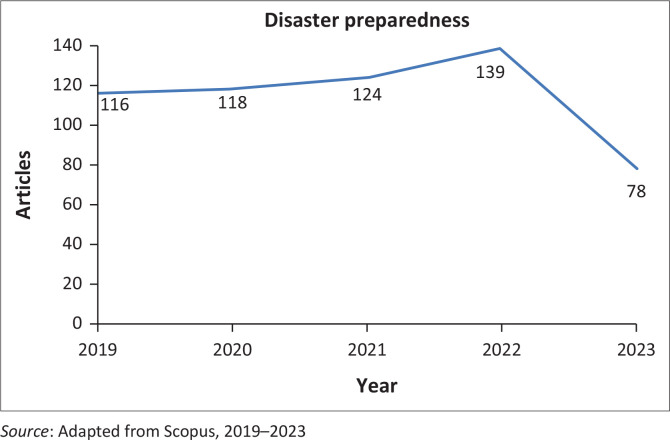
Trend in disaster preparedness research yearly.

Figure 2 indicates the trend of disaster preparedness research based on the number of articles published yearly. Generally, research on disaster preparedness in the world is expanding in 2019 with 116 publications, demonstrating an increasing number of articles published each year associated with disaster risk mitigation strategies and techniques. The journal covers a wide scope of topics regarding disaster preparedness and has steadily increased publications since 2019 (Moral-muñoz et al. 2020; Oktari 2022; Sakurai & Murayama 2019). The data analysis that was published up to 2023 was completed with bibliometrics to understand the research trends.

**FIGURE 3:**
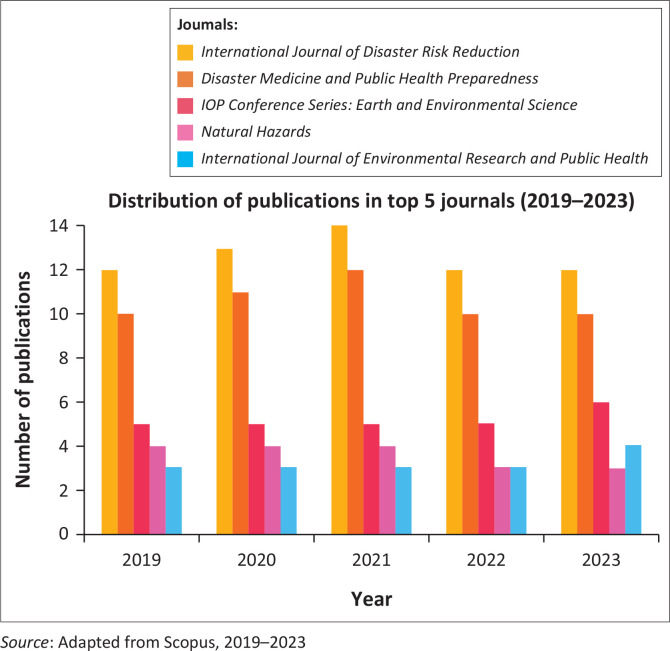
Top five publishers of disaster preparedness journals.

Figure 3 displays the top five peer-reviewed journals with the most publications in disaster preparedness research, with a total of 575 documents analysed from 2019 to 2023.

**FIGURE 4:**
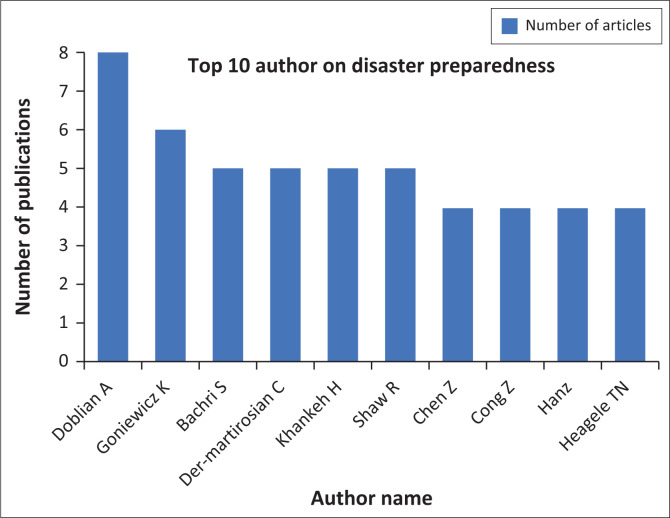
Top 10 authors of disaster preparedness articles.

Figure 4 shows the top 10 authors in the field of disaster preparedness with the highest number of publications during the 2019–2023 period indexed in Scopus. Each author is expected to have published between 2 and 4 publications. Notably, Dobalian, A. holds the highest position with 8 publications concerning disaster preparedness. Goniewicz, K. follows with 6 publications, while Bahri, S., Der-Martirosian, C. and Khankeh, H. have 5 publications each. These results demonstrate the significant contributions of these authors in disaster preparedness research.

**FIGURE 5:**
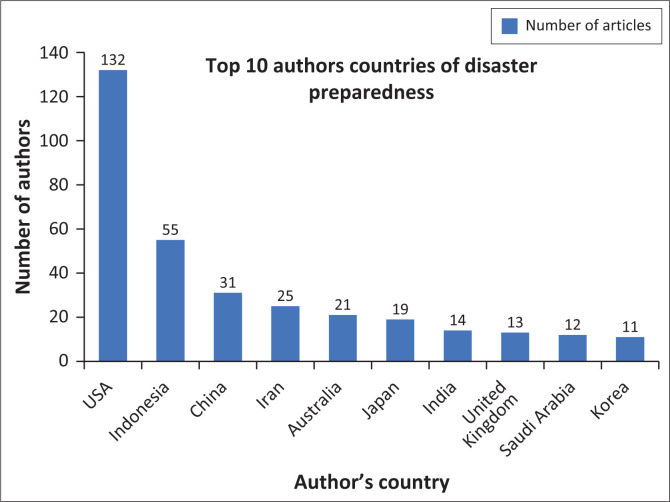
Top 10 authors countries of disaster preparedness.

Figure 5 reveals the contribution of authors from several countries to the research on disaster preparedness. The United States leads with 132 authors, which is followed by Indonesia with 55 authors. China has 31 authors, while Iran has 25 authors. The other countries including Australia, Japan, India, the United Kingdom, Saudi Arabia and South Korea also exhibited active participation, with 21, 19, 14, 13, 12 and 11 authors, respectively. These data reflect the wide geographical spread of disaster preparedness research around the world.

**FIGURE 6:**
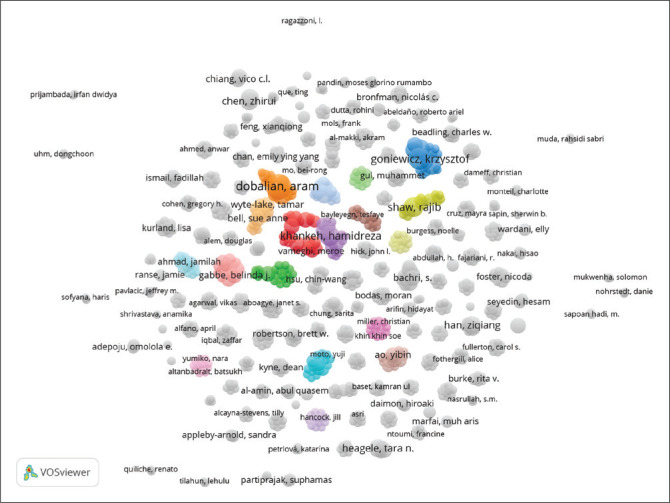
Collaborative network within researchers on disaster preparedness topics.

Figure 6 is a visualisation of keywords related to the disaster preparedness research topic labelled and denoted as circles by default in VOSviewer. The more commonly the word appears, the larger the circle. The size of the circle indicates the importance of a keyword. The more prominent an item or keyword is, the more frequently it appears, so the larger the circle. The frequency of occurrence determines the size of the item. This keyword is particularly important as it indicates that the more frequently the word appears, the larger the circle. This figure denotes the keywords that have the primary research topics (main topics). The most prominent and primary topics involved ‘disaster preparedness’, ‘disaster prevention’, ‘natural hazard’, ‘human experiment’, ‘cross-sectional study’ and ‘questionnaire’. These terms frequently appear and have a lot of links to other topics, indicating their importance in the field of disaster preparedness research.

Figure 6 highlights the result of the collaborative network among researchers. Table 3 shows the result of a cluster analysis based on the frequency of keywords and the most frequently emerging keywords.

**FIGURE 7:**
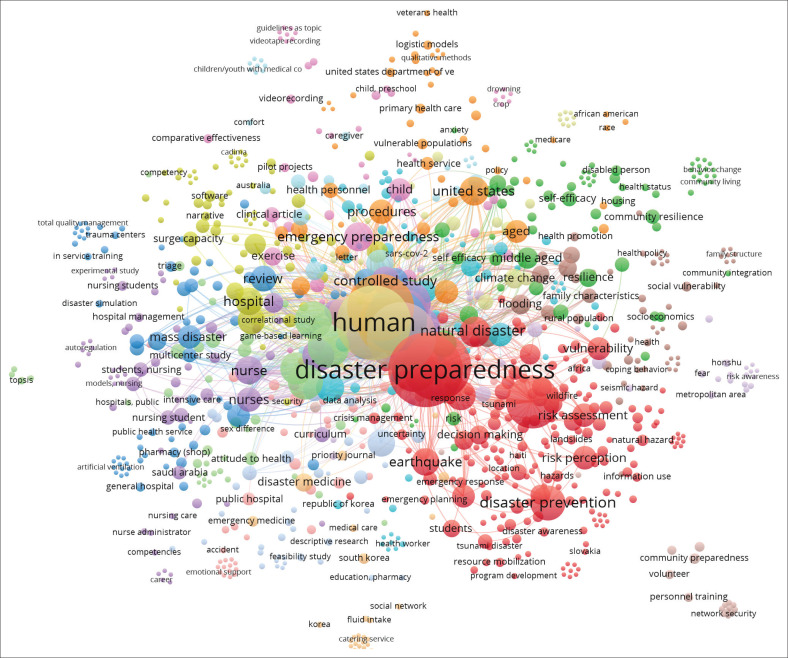
Trending topic of disaster preparedness.

In addition, the results of Figure 7 are trending topics about disaster preparedness. Where it shows the visualization of the collaboration network between researchers based on citations using VOSviewer software. Each point (node) represents an author or article, and the size of the node reflects the frequency of citations. The lines connecting the nodes indicate collaborations or relationships between the co-citation analysis of authors in scientific articles.

In this figure, the names of authors displayed with a larger node indicate that they are highly cited authors or articles. Some of the authors that seem to be dominant include the following: Dobalian, Aram (located in the centre of the network with a large node, signifying that the works of this author are frequently cited by other researchers); Khankeh, Hamidreza (this author also has a large node, marking a significant influence in the study); Gabbe, Belinda J. (another clearly visible name with a large node, representing a high citation rate).

The authors are in the middle of particular colour clusters, indicating that they have strong collaboration networks and are frequently referenced in research. Clusters of different colours refer to interrelated research clusters or topics. Collectively, this visualisation reveals that authors including Dobalian, Aram, Khankeh, Hamidreza, and Gabbe, Belinda J. are the most influential authors in this network, based on the number of citations.

**FIGURE 8:**
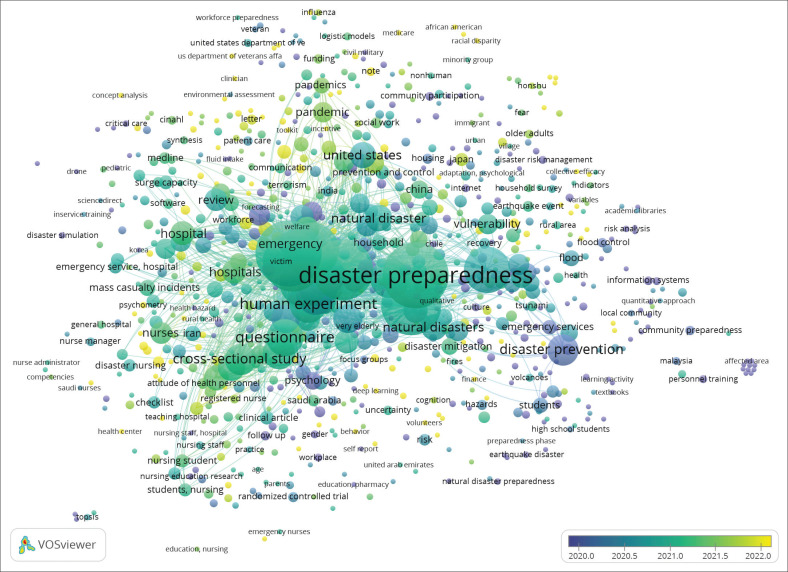
The evolution of research methodology.

Based on Figure 8, which visualises the evolution of research methodologies in disaster preparedness studies, it can be seen that the research approaches have evolved over time. In 2019, survey and observational methods were the dominant approaches in collecting data regarding disaster preparedness. In 2021, research began to employ more qualitative approaches and longitudinal studies to get an in-depth understanding of the phenomenon. In 2022, there was an emphasis on the use of experimental methods and experimental studies to test the hypotheses in a more controlled method. In 2023, there was a shift towards big data analysis and multidisciplinary approaches, which enabled a more comprehensive and integrative understanding of disaster preparedness studies. This evolution reflects progress and diversification in research approaches as issues regarding disaster preparedness become more complex.

**FIGURE 9:**
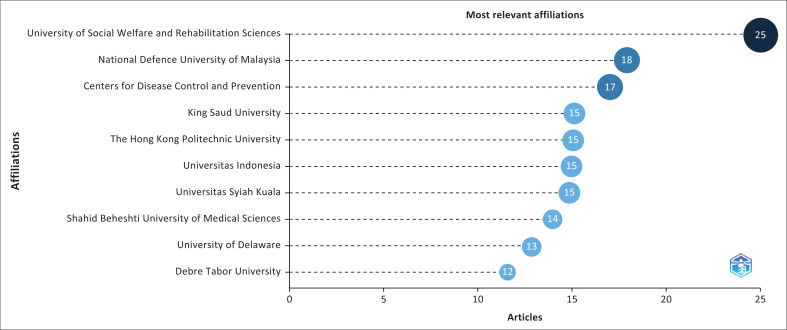
The most relevant affiliations.

Figure 9 demonstrates the institutions or organisations that most frequently appear in disaster preparedness publications. The ‘Most Relevant Affiliations’ graph that was generated by Biblioshiny revealed that the University of Social Welfare and Rehabilitation Sciences had the largest contribution with 25 articles. The other notable institutions involved the National Defence University of Malaysia with 18 articles; the Centre for Disease Control and Prevention with 17 articles; and King Saud University, The Hong Kong Polytechnic University, Universitas Indonesia and Universitas Syiah Kuala, which contributed 15 articles each. Shahid Beheshti University of Medical Sciences provided 14 articles, closely followed by the University of Delaware with 13 articles, and Debre Tabor University with 12 articles. These data highlight the essential role played by diverse institutions in disaster preparedness research.

## Discussion

Globally, the implementation of bibliometric methods in disaster preparedness proposes a systematic and database approach to enhance the understanding of disaster risk mitigation strategies, disaster medicine preparedness and public health, and bibliometric earth and environmental sciences (Donthu et al. 2021; Hoque et al. 2022; Molassiotis et al. 2021; Rana 2022). The analysis of 575 articles was classified into 10 subtopics see Table 2, and the emerging topics included community-based disaster risk mitigation strategies, the integration of public health in hazard preparedness, and the fundamental role of earth and environmental sciences in mitigating natural hazard such as earthquakes and floods. In addition, topics such as sustainability and medical responses to disasters were also an essential focus in the literature reviewed, with an emphasis on the contribution of mitigation strategies to sustainable development and effective health responses in emergency situations (Kramer 2017; Le Duff et al. 2020; Zuccaro et al. 2020).

One of the main indicators of this research trend is the increasing quantity of publications in reputable journals related to disaster preparedness, demonstrating an elevated number of publications yearly, thereby pointing to the heightening of research interest (Hargono et al. 2023; Verheul & Dückers 2020). The *International Journal of Disaster Risk Reduction* has the highest publication rate with 63 articles, demonstrating its dominant role in supporting research in disaster preparedness. The other journals such as *Disaster Medicine and Public Health Preparedness* and *IOP Conference Series: Earth and Environmental Science* consistently publish numerous articles each year with 53 and 26 publications in each see Table 1.

**TABLE 1:** Top 10 journals with the most publications.

Journal name	Ranking	Frequency
International Journal of Disaster Risk Reduction	1	63
Disaster Medicine and Public Health Preparedness	2	53
IOP Conference Series: Earth and Environmental Science	3	26
Natural Hazards	4	18
International Journal of Environmental Research and Public Health	5	16
Jamba: Journal of Disaster Risk Studies	6	8
Sustainability (Switzerland)	7	8
Disasters	8	7
Journal of Physics: Conference Series	9	7
American Journal of Disaster Medicine	10	6

Note: As can be seen from Table 1, International Journal of Disaster Risk Reduction is ranked first in the publication of disaster preparedness topics with 63 documents. Disaster Medicine and Public Health Preparedness is ranked second with 53 documents. The journal IOP Conference Series: Earth and Environmental Science is ranked third with 26 documents, Natural Hazards is ranked fourth with 18 documents, and International Journal of Environmental Research and Public Health is ranked fifth with 16 documents.

Table 1 indicates the published articles based on the 10 journals with the highest publications in the disaster preparedness domain from 2019 to 2023.

**TABLE 2:** Summary of general information.

Description	Results
**Main information about data**	
Timespan	2019–2023
Sources (journals, books, etc.)	288
Documents	575
Annual growth rate (%)	10.94
Document average age	3.16
Average citations per document	10.17
References	22 769
**Document contents**	
Keywords plus (ID)	1870
Author’s keywords (DE)	1223
**Authors**	
Authors	2026
Authors of single-authored documents	57
**Authors collaboration**	
Single-authored documents	59
Co-authors per document	3.99
International co-authorships (%)	24
**Document types**	
Article	416
Book	1
Book chapter	16
Conference paper	56

**TABLE 3:** Cluster analysis based on keywords.

No	Item	Description
1	Cluster identification	The image indicates several researcher clusters represented by different colours. Each cluster shows a group of researchers who frequently collaborate or have strong relationships in their research.
2	Clusters with high frequency	Pink cluster (Hamidreza Khankeh, Belinda J. Gabbe, Jamie Ranse): Researchers in this cluster frequently use keywords associated with health topics, particularly those related to emergency services and disaster management. Blue cluster (Aram Dobalian, Gregory H. Cohen, Tamar Wyte-Lake): This cluster involved research in public health and health policy. Yellow cluster (Krzysztof Goniewicz, Muhammad Gul): The researchers in this cluster tend to focus on the topics of disaster management and emergency preparedness. Green cluster (Ziqiang Han, Yibin Ao): The studies in this cluster are primarily concerned with infrastructure and engineering topics.
3	Frequently appearing keywords	‘Disaster Management’ and ‘Emergency Services’: Frequently appear in pink and yellow clusters. ‘Public Health’ and ‘Health Policy’: Dominant in blue clusters. ‘Infrastructure’ and ‘Engineering’: Appear in green clusters.
4	Interaction in cluster	Some researchers at the edge or centre of some clusters exhibit collaboration within different topics and indicate multidisciplinary research.

The data reveal a significant increase in the number of annual publications, indicating a renewed global interest in disaster preparedness research. This increase is also driven by various factors, including the increasing frequency of natural hazard and their mitigation (Nousheen et al. 2020). These journals not only note the number of articles published but also exhibit the diversity of topics covered, including aspects of public health, earth sciences and the environment (Lee et al. 2020; Zhang et al. 2021).

As a result, the *International Journal of Disaster Risk Reduction* occupies the first position as the journal that most extensively publishes research regarding disaster preparedness, with a total of 63 publications. This journal has been a prominent reference in disaster risk mitigation research, hence being an essential reference for researchers and practitioners in this discipline. The second position is occupied by *Disaster Medicine and Public Health Preparedness*, which focuses on medical and public health preparedness, with 53 publications. These two journals played a significant role in steering academic and practical field attention towards effective strategies for dealing with disasters.

In addition to these two headline journals, the *IOP Conference Series: Earth and Environmental Science* with 26 publications and *Natural Hazards* with 18 publications are also prominent in the research associated with disaster. These journals examine broad aspects of earth and environmental science, including how natural hazards can be mitigated through scientific and strategic approaches. The publications in these journals demonstrate the importance of a better understanding of the physical environment to enhance preparedness and response to natural hazards.

The other journals with significant contributions included the *International Journal of Environmental Research and Public Health*, which highlights the interaction between the environment and public health, and *Jamba: Journal of Disaster Risk Studies*, which examines the various aspects of disaster risk studies. Journals such as *Sustainability (Switzerland)* and *Disasters* also play a role in exploring sustainability and disaster mitigation, while *Journal of Physics: Conference Series* and *American Journal of Disaster Medicine* provide insight into disaster risk from a physics and medical perspective. The combination of these journals establishes a strong foundation for better disaster preparedness research and practice in the future.

The contribution of authors in the field of disaster preparedness is an essential aspect that deserves extra attention in literature analysis (Coffey et al. 2021). One of the prominent authors is Aram Dobalian, who is listed as the author with the highest number of publications, which is 8 documents. Through his works, Dobalian has made significant contributions for enriching knowledge and advancing scientific discussions concerning disaster preparedness. Dobalian’s publications not only contribute to the volume of literature but also proffer deep insights into effective strategies in confronting and managing disaster risks.

Besides Dobalian, Krzysztof Goniewicz and Bahri S. are also impactful authors in disaster preparedness research, with six and five published papers, respectively. Their works have strengthened the knowledge base in this field, aiding the academic and practitioner communities to better understand the challenges and solutions pertaining to disaster preparedness. Generally, the contributions of these authors not only broadened the existing literature but also encouraged the development of more effective strategies for disaster mitigation and response around the world.

The United States stands out as the country with the largest contribution to disaster preparedness research, producing 132 publications that reinforce its position as a global leader in this field. This significant contribution demonstrates the country’s determination to develop effective strategies for dealing with different types of natural hazards, given the frequent occurrence of disasters in its region. In addition, the United States is also a global centre for international collaboration, encouraging knowledge exchange and innovation in disaster risk management.

Indonesia stands in second place with 55 publications, followed by China with 31 publications. These two countries, also vulnerable to numerous natural hazards, demonstrate a solid commitment to disaster preparedness research. Their contributions are not only significant for global scientific development but also crucial for mitigation strategies in their respective regions. In general, the contributions from these countries emphasised a persistent global commitment to enhance disaster preparedness and response, prompted by the immediate need to address the evolving threat of natural hazards.

The United States remains as the most active country in publishing research regarding disaster preparedness, not only because of the high volume of publications but also because of its powerful international collaboration network. Researchers from the United States consistently collaborate with colleagues from other countries, such as the United Kingdom, China and Australia. This collaboration creates synergies that enrich research and result in more comprehensive strategies for addressing a variety of natural hazards.

This international collaboration network plays an essential role in accelerating the exchange of knowledge and innovation in the disaster preparedness field. Through these collaborations with researchers from various countries, the United States has successfully integrated diverse perspectives and expertise, which in turn strengthens global opportunities to develop more effective disaster mitigation and response strategies. These collaborations not only elevate the quality of research but also contribute to improved disaster preparedness on a global scale.

**FIGURE 10:**
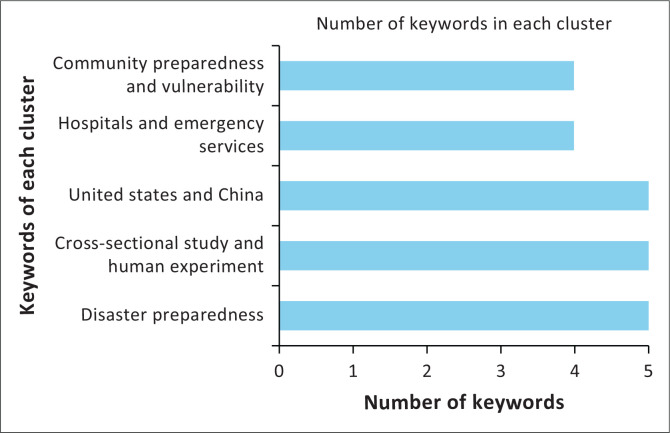
Number of keywords for each cluster.

This research identifies five primary clusters in disaster preparedness analysis based on the frequency of keyword associations. The primary cluster, ‘Disaster Preparedness’, focuses on disaster preparedness, incorporating prevention aspects and the fundamental role of hospitals and emergency services. The second cluster, involving ‘Cross-Sectional Study’ and ‘Human Experiment’, highlights the significance of cross-sectional studies and human experiments, particularly in understanding the role of nurses in disaster preparedness. The other clusters, which include ‘United States’ and ‘China’, mainly concentrate on geographical research pertaining to disasters in both countries. Meanwhile, the ‘Hospitals’ and ‘Emergency Services’ clusters emphasise the important role of hospitals and emergency services in managing critical situations. The last cluster, ‘Community Preparedness’ and ‘Vulnerability’, underlines community preparedness and the psychological impact and social vulnerability in the face of disasters see Figure 10.

This research has significant implications for boosting disaster preparedness in various sectors. Recognising the patterns and focuses of the identified research, decision-makers can effectively design a comprehensive data-driven disaster prevention strategy. This may include optimising the roles of hospitals and emergency services and augmenting community preparedness by considering social vulnerability and potential psychological impacts. Additionally, these findings provide valuable insights for academics and health care practitioners to develop further studies that align with global needs in disaster preparedness, ensuring that the strategies developed are relevant and applicable across different contexts (Khairina, Nelwati & Ariany Maisa 2022; Pickering et al. 2021).

In the co-occurrence network analysis, Biblioshiny highlights keywords that co-occur frequently, while VOSviewer provides a visual network of associated keywords. This enables the identification of gaps among the main research trends, which can serve as the foundation for possible new studies. Additionally, citation analysis with Biblioshiny highlights the most commonly cited articles, while VOSviewer visualises co-citation relationships, assisting in identifying foundational literature and uncovering research gaps. In terms of theme evolution, Biblioshiny tracks changes in keyword trends over the years, and VOSviewer visualises topic development, revealing emerging areas that are beginning to attract attention in research and adding a dimension of novelty to this study see Table 5.

In disaster preparedness research, the most impactful topics involve ‘disaster preparedness’, ‘disaster prevention’ and ‘natural disaster’. These keywords frequently appear in the literature, implying that they are the primary focus of a lot of current studies. The high frequency of these keywords indicates significant attention from the scientific community on the importance of disaster preparedness and prevention. The emphasis on these topics highlights ongoing attempts to understand and develop strategies that can mitigate vulnerability and the risks associated with natural hazards.

The overwhelming preference for disaster preparedness and prevention in the scientific literature underscores that these strategies are deemed essential in minimising the negative impacts of natural hazards. Effective preparedness enables communities and institutions to quickly and efficiently respond to disasters, while prevention focuses on mitigating the likelihood of disasters occurring or reducing their impact. Therefore, the research centred on these topics makes a substantial contribution to the development of better policies and practices in disaster risk management and supports global attempts to enhance disaster resilience at various levels.

Trending topics in disaster preparedness research nowadays include emergency services, the role of hospitals and cross-sectoral studies. The focus on these topics exemplifies the increasing emphasis on medical preparedness, public health and the importance of resilient infrastructure in disaster scenarios. The research in this scope highlights the way in which the readiness of hospitals and the efficiency of emergency services can determine the success of disaster response and the way in which cross-sectoral approaches provide a more comprehensive understanding of disaster preparedness see Table 4.

**TABLE 4:** Cluster analysis results with ‘number of keywords’.

Cluster	Main keywords	Number of keywords
Main keyword: ‘Disaster Preparedness’	disaster preparedness, natural hazard, emergency services, disaster prevention, hospitals	5
Supporting cluster: ‘Cross-Sectional Study’ and ‘Human Experiment’	cross-sectional study, human experiment, questionnaire, nurses, nursing.	5
Geographic cluster: ‘United States’ and ‘China’	United States, China, pandemics, flood, earthquake	5
Health Cluster: ‘Hospitals’ and ‘Emergency Services’	hospitals, emergency services, critical care, mass casualty incidents	4
Social Cluster: ‘Community Preparedness’ and ‘Vulnerability’	community preparedness, vulnerability, social work, psychological impact	4

**TABLE 5:** The updated disaster preparedness mapping analysis.

Aspects of analysis	Results from Biblioshiny	Results from VOSviewer	Novelty
Keyword trends	Identification of dominant keywords	Visualisation of association within keywords	Revealing new keywords that have not been extensively discussed
Co-occurrence network	Frequent keywords	Visual network of the relevant keywords	Identifying gaps within the main research trends
Citation analysis	The most frequently cited articles	Visualisation of co-citation correlations	Discover the basic literature and identify the research gaps
Theme evolution	Changes the keyword trends from year to year	Visualisation of topic evolutions	Discover new emerging areas of research interest

Additionally, some topics related to human experimentation are also gaining significant attention, reflecting a growing interest in understanding how individuals and communities respond to disasters. This research is fundamental to develop more effective mitigation and response strategies, because it can reveal psychological and social factors that influence human behaviour in emergency situations. In understanding these responses, researchers and practitioners can design more appropriate interventions, enhancing community preparedness and resilience against future disasters.

Trend analysis of disaster preparedness research trends reveals that keywords such as ‘disaster preparedness’, ‘disaster prevention’, ‘natural hazard’ and ‘emergency’ are the most dominant keywords. These trends highlight a persistent focus on disaster preparedness and prevention in recent literature, with these topics being discussed intensively from 2020 to 2022. ‘Disaster preparedness’ emerges as the most prominent keyword, underscoring the importance of this concept in research pertaining to disaster mitigation and response.

The inherent connection within keywords such as ‘disaster preparedness’, ‘natural hazard’ and ‘emergency services’ indicates that disaster preparedness research is often directly linked to disaster response aspects. The network visualisation also reveals the complexity and interconnection among the various topics in this study. However, emerging topics like ‘psychology’, ‘vulnerability’, ‘community preparedness’ and ‘climate change’ are beginning to gain attention, although their connections in the network are marginal, showing that these topics have potential for further research.

In addition, papers with keywords such as ‘disaster preparedness’ and ‘disaster prevention’ seem to be the most frequently cited, demonstrating their significant influence in the disaster preparedness literature. The co-citation relationships within the main studies, such as those related to ‘emergency services’ and ‘natural hazards’, suggest that these studies are often referenced together, forming an interconnected knowledge base that supports the development of more comprehensive strategies for disaster mitigation and response.

Potential future topics in disaster preparedness research involve ‘community preparedness’, ‘vulnerability’ and ‘climate change’. Although these topics are not currently widely explored, they are demonstrating increasing interest and relevance, particularly as global challenges like climate change and social vulnerability continue to evolve (Le Quéré et al. 2020). Extended research in these areas is necessary to develop more inclusive and sustainable preparedness strategies.

Within the scope of disaster preparedness research, the most challenging future topics involve ‘community preparedness’, ‘vulnerability’ and ‘climate change’ (McKenney & Reeves 2018; Rasheed et al. 2021; Righi, Saurin & Wachs 2015). While these topics may not currently be widely explored, they are showing significant growth in interest and relevance, particularly as global challenges like climate change and social vulnerability continue to evolve. For instance, ‘community preparedness’ focuses on measuring whether communities can effectively prepare for disasters by enhancing capacity and collective participation (Chari et al. 2019). Similarly, ‘vulnerability’ examines the degree to which individuals and communities are susceptible to the disaster impacts, which are influenced by social, economic and environmental factors. This topic is increasingly crucial, given the vulnerability disparities among different social groups in facing disasters.

Considerable research on ‘climate change’ with regard to disaster preparedness is also necessary, especially considering the increasingly evident impact of climate change on the frequency and intensity of disasters (Le Quéré et al. 2020; Ma et al. 2021). Climate change has the potential to exacerbate existing vulnerabilities and create new challenges for preparedness strategies. Therefore, integrating climate change research with disaster preparedness studies is crucial for developing more inclusive and sustainable strategies. This effort would not only reinforce preparedness at the local level but also significantly contribute to broader mitigation policies and actions on a global scale.

The ‘Most Relevant Affiliations’ graph from Biblioshiny highlights the most active institutions in publishing research on the analysed topic, in which the University of Social Welfare and Rehabilitation Sciences stands out as the primary contributor with 25 articles. This dominance indicates that the institution probably has significant resources, researchers and projects in this discipline, qualifying it as a prominent research centre. The identification of these outstanding institutions helps focus present research and provides insights into future trends. Additionally, the data reveal the potential for collaboration among institutions with similar publication numbers, which could reinforce research networks and accelerate progress in the field. The other institutions can employ this information to form strategic partnerships or explore popular topics, enhancing their own research and contributions to the global scientific literature.

The methodology in disaster preparedness research has evolved significantly over time, which reflects the necessity to understand and address increasingly complex challenges in the field. In 2019, the most commonly adopted methods were surveys and observations, which provided a fundamental overview of disaster preparedness through direct data collection from respondents and real-world observations. However, along with the increasing demand for deeper analysis, research in 2022 started to shift in favour of longitudinal and experimental studies. These approaches enabled researchers to track changes in disaster preparedness over time and identify factors that contribute to the effectiveness or weaknesses of the strategies employed. Particularly, longitudinal studies provide insights on how responses and preparedness evolve over time, while experiments permit the testing of specific interventions to assess their impact under controlled conditions.

In 2023, research methodology has increasingly evolved with the implementation of big data analysis and multidisciplinary approaches, reflecting the increasing complexity of challenges faced in disaster preparedness. The employment of big data enables researchers to analyse large amounts of information covering different aspects of preparedness, such as weather patterns, data population and disaster responses, on a significantly larger scale than previously possible (Cutter & Derakhshan 2020). On the other hand, multidisciplinary approaches integrate different fields of study, including public health, engineering, social sciences and environmental science, to create more holistic and effective strategies for disaster management. This shift marks a significant transformation in disaster preparedness research, where more sophisticated and comprehensive approaches are needed to address the complexities of a changing world (Elamin et al. 2018; Nappo et al. 2021).

## Conclusion

The research on disaster preparedness has exhibited a significant upward trend in the number of publications each year, reflecting the global interest in this topic. The United States stands out as the most active country in this field, with the largest contribution and a broad collaboration network with countries like the United Kingdom, China and Australia. Several journals, such as the *International Journal of Disaster Risk Reduction* and *Disaster Medicine and Public Health Preparedness*, have been the primary platforms for publishing numerous articles on disaster preparedness.

The primary topics frequently discussed in the literature involved ‘disaster preparedness’, ‘disaster prevention’ and ‘natural hazard’, which were the core of recent studies. The research also pointed emerging topics that could be a future point, such as ‘community preparedness’, ‘vulnerability’ and ‘climate change’. While these topics are currently less explored, they are gaining increased attention because of their growing relevance in the context of global climate change and social vulnerability challenges.

The research methodology in this field has evolved from survey and observational methods to longitudinal studies, experimental designs and big data analysis, reflecting the increasing complexity of understanding disaster preparedness. Some institutions like the University of Social Welfare and Rehabilitation Sciences played a significant role in advancing research in this discipline, highlighting the importance of collaboration among institutions developing more comprehensive and sustainable preparedness strategies.

### Limitations

The main limitation of this study is the use of data from scientific papers that are only found in specific databases; as a result, the result of the analysis’s findings may not encompass the full range of studies conducted in the field of disaster preparedness. In addition, this study makes extensive use of bibliometric methodology, which has limitations in capturing the finer details and context of individual studies, including the quality of research, the methods used and the relevance of each paper’s local context, regardless of whether it provides a systematic and data-driven view. Another limitation is the focus placed on countries with a high number of publications, which may overlook the important contribution of research conducted in countries with more limited resources but with direct experience of disasters. In addition, topics that are only just beginning to receive attention such as ‘community preparedness’ and ‘vulnerability’ may be under-represented in this analysis, and their potential importance in the development of future preparedness strategies may be overlooked.
